# London Post-Graduate Institutions

**Published:** 1909-09-04

**Authors:** 


### LONDON POST-GRADUATE INSTITUTIONS.

### WEST LONDON POST-GRADUATE COLLEGE.

The West London Hospital, Hammersmith, was the first of the general hospitals in London to reserve its practice strictly for qualified men, and to establish a post-graduate College as an integral part of its constitution. Post-graduate instruction has been given at the hospital since the year 1893, and over 1,477 medical men from all parts of the world have attended the classes up to December 31, 1908. Of these 413 have been officers in the Naval, Army and Indian Medical Services, and 367 others have come from the Colonies and foreign countries. The hospital itself contains 160 beds, and the advantages of the direct connection of the College with the wards and out-patient work are obvious. The hospital is recognised by the Royal College of Surgeons as an institution where candidates (who need not be members) for the Fellowship can spend the necessary year of study after obtaining a registrable qualification, while the College and hospital are also recognised by the naval and military authorities as regards study leave. The hospital is authorised by the University of London to give certificates of post-graduate study for the M.D. and M.S. degrees.

Members of the College may also accompany the resident staff on their visit to the wards, and so have opportunities of refreshing and extending their knowledge of clinical methods. Operations are performed daily, at 2.30 p.m. Post-graduates are allowed to stand near the table and can see the operations perfectly. The surgeons often avail themselves of the assistance of post-graduates at operations. In the theatre post-graduates may, under the direct supervision of the anæsthetists of the staff, administer anæsthetics in major operations.

The out-patient departments necessarily resemble in their main features those of other general hospitals, and the staff are always alert to discover, and, when found, to demonstrate, any cases of exceptional interest, or, what is of equal importance, any points of special interest found in cases of the more common diseases. During the sessions lectures are given daily, except Saturdays, at 5 p.m., on practical medicine and surgery in all their branches. There are also short courses on tropical diseases, and on mental diseases by men who have made their mark in those subjects. These lectures are quite distinct from the clinical demonstrations in the wards by the physicians and surgeons, and may be attended without additional fee.

Small classes are formed, when required, in connection with diseases of special regions, such as eye, throat, skin, etc.; for the clinical examination of blood and urine; in bacteriology and medical microscopy generally; in applied anatomy, administration of anæsthetics, intestinal surgery, cystoscopy, x-ray work, etc. For these classes, each of which is strictly limited to a small number, additional fees are required, as they are practically individual and tutorial. The ordinary fee for membership covers attendance at all general lectures and at all the routine practice of the hospital, whether in the general or special wards or departments. Autopsies can, of course, be attended. Arrangements can be made through the College authorities for special coaching should it be desired.

Rooms are provided for members where they may read, write, and smoke, and consult books of reference; tea can be obtained in the reading-room; lockers are provided at a small rent for those who wish to keep books or such articles at the College. The medical library of the West London Medico-Chirurgical Society is housed in the College, and post-graduates have the use of the books and periodicals. The hospital is very accessible. Good lodgings are obtainable in the neighbourhood, a list of addresses being kept by the Secretary of the College.

Members may join for any period from one week upwards, the fees ranging from one guinea to £30. Some few years ago it was considered expedient to increase the fee for life membership, and this enhancement was carried out and remains in force without any resulting diminution of membership.

### THE LONDON POLYCLINIC.

The Medical Graduates' College and Polyclinic has now a well-recognised position among the medical educational organisations of the metropolis. Its object is to provide opportunities for clinical and practical study for those who have already gained a position on the medical register. From the outset two principles have been recognised as essential to a large and broad scheme of post-graduation study. First, that such a scheme should be entirely separate and distinct from the ordinary educational course of the medical student. Secondly, that the teaching should not be confined to the staff of any individual hospital, but should include capable and distinguished representatives of all schools. Hence, at the Polyclinic, the needs of the practitioner receive full consideration, and the teaching in a very special manner represents the work of many of the best-known physicians and surgeons in the metropolis.

The afternoon cliniques are conducted daily, and offer valuable opportunities. Medicine, surgery, and each of the more special branches of practice, has its appropriate day. Selected cases are demonstrated and made the subject of clinical comment, and opportunities for personal examination by those attending the clinique are offered. In this way, and in a comparatively short period of time, the practitioner is able to overtake a large quantity of interesting and instructive clinical material under the direction of teachers specially qualified in each of the various departments. Indeed, it is difficult to imagine any scheme more suitable for those who are anxious to increase their clinical experience both in general and special work. On four days of each week there is also a lecture by a teacher from a provincial or a metropolitan school. In these lectures the practical note is maintained throughout.

With a view to render these opportunities accessible to all, the annual subscription, which includes admission to all cliniques and lectures and also the use of the library and museum, has been fixed at one guinea. In addition, the *Polyclinic Journal* is issued monthly, and is sent free to all subscribers; and in the College laboratory specimens are examined and reported on for small fees. Another part of the Polyclinic scheme, and one of great importance, is the provision of "practical classes" in the more recent methods of clinical investigation. Included in this division, instruction is provided in the use of the ophthalmoscope, laryngoscope, otoscope, and other specialised clinical instruments; in the clinical examination of the blood, sputa, urine, etc.; in the use of the x-rays; and in practical gynæcology. In each class the numbers are limited. A special vacation course of these practical classes is to commence on Monday, September 13, and is completed by October 1.

Tutorial classes for practitioners reading for the higher examinations have recently been instituted, and have proved most successful. Particulars as to fees, hours, etc., can be obtained upon application to the Medical Superintendent, 22 Chenies Street, Gower Street, W.C.

The success already achieved by the Polyclinic is a considerable one, and it appears to have a future of still greater usefulness before it.

### THE LONDON SCHOOL OF CLINICAL MEDICINE (POST-GRADUATE).

The Seamen's Hospital, Greenwich.

The wise and enlightened policy followed by the Board of Management of the Seamen's Hospital Society in deciding to render available for clinical post-graduate study the wards, theatres, etc., of their parent hospital at Greenwich, as they had already thrown open their branch hospital at the Albert Dock for the study of tropical diseases, has already fully justified itself by results. It has proved that in addition to the considerable facilities already afforded for post-graduate study in London, there existed a demand for opportunities of clinical investigation and teaching untrammelled by the presence of undergraduates, greater than that already supplied by the hospitals engaged in this special work.

Since the doors of this hospital were thrown open to graduate students in 1906 some hundreds of men have passed through courses of instruction within its wards—men drawn from all parts of the world, from private practice and from the medical services. The greater part of the clinical teaching of this School is given at the Seamen's Hospital, Greenwich, but in addition, for the purpose of making the curriculum more comprehensive, the Royal Waterloo Hospital for Children and Women, the Bethlem Hospital for Mental Diseases, and the York Road Lying-in Hospital have been affiliated for teaching purposes; while recently the authorities of the Royal Eye Hospital, Southwark, have thrown open their doors to students from the School of Clinical Medicine under specially advantageous terms of preference. These hospitals are all situated on the south side of the Thames, and are linked to one another by tram and rail, so that post-graduates attending the services of the new School are enabled to take out courses of instruction in every department of medicine and surgery. The certificates of the School are recognised by the Admiralty, the War Office, the Colonial Office, the University of London, and other educational bodies.

The *Dreadnought* Hospital, which is the centre of the organisation, is situated at Greenwich, close to both railway stations, and within thirty to forty minutes' reach of London by rail or tram. The hospital contains 250 beds, and these are continuously occupied by cases of wide and varying clinical interest. The admissions to the *Dreadnought* Hospital differ from those at most other hospitals, in that cases of tuberculosis and venereal disorder are admitted. The wards are mostly small, many of them containing no more than three beds. There are, therefore, unusual opportunities for the individual examination of cases.

The out-patient department has been reorganised and equipped with the latest modern requirements. Besides the ordinary medical and surgical consulting-rooms, it provides special departments for diseases of the eye, diseases of the skin, and diseases of the ear, throat, and nose. There is a large and admirably-arranged operating theatre for in-patients, and a smaller one in the out-patient department. There are also two laboratories, pathological museum, and post-mortem rooms, where pathology and operative surgery are taught. In the pathological department arrangements have been made whereby investigations of all sorts—chemical, microscopical, biological, etc.—are undertaken at moderate fees. For the purposes of the School there are lecture rooms and a library, while the comfort of those who attend the classes has been kept in view by the provision of comfortable reading and smoking rooms. Men engaged in practice in the neighbourhood of the hospital have been invited to join the School as associates at a nominal fee, and a considerable number have already availed themselves of the privilege. By this plan it is possible for those within reach to visit the hospital and attend the cliniques when it is convenient for them to do so—a most excellent arrangement for busy men.

### NORTH-EAST LONDON POST-GRADUATE COLLEGE.

This School, which is in connection with the Prince of Wales's General Hospital, Tottenham, N., is recognised by the University of London, the Admiralty, and the India Office as a place of post-graduate study. Opportunities are here afforded for attending demonstrations on various branches of medicine, surgery, and gynæcology, with opportunities for clinical instruction in diseases of the eye, ear, throat, nose, skin, and in fevers, diseases of children, psychological medicine, anæsthetics, *x*-rays, bacteriology, and dentistry. Special classes with a limited attendance have been arranged for these and other subjects. The fee for a three-months' course in any single department is one guinea, or three guineas admits for a similar term to the whole practice of the hospital. A perpetual ticket costs five guineas. Additional information, with syllabus of lectures and special classes, from the Dean, Dr. A. J. Whiting, 142 Harley Street, W.

### OPPORTUNITIES FOR GRADUATE STUDY IN SPECIAL HOSPITALS.

The London Post-Graduate Association.

